# How do the post-graduation outcomes of students from gateway courses compare to those from standard entry medicine courses at the same medical schools?

**DOI:** 10.1186/s12909-023-04179-3

**Published:** 2023-05-02

**Authors:** Ahmad Elmansouri, Sally Curtis, Ceri Nursaw, Daniel Smith

**Affiliations:** 1 Medical Education, Faculty of Medicine, University of Southampton, Southampton, SO17 IBJ England UK; 2 Nursaw Associates, Coventry, CV1 2TT England UK; 3 General Medical Council, London, NW1 3JN England UK

**Keywords:** Education, Medical, Postgraduate, Widening participation, Attainment, Outcomes, Progression, UKMED, Gateway

## Abstract

**Background:**

Widening participation (WP) for underrepresented students through six-year gateway courses helps to widen the demographic representation of doctors in the UK. ‘Most students from gateway courses graduate, even though many enter with lower grades than standard entry medicine students.’ This study aims to compare the graduate outcomes of gateway and SEM cohorts from the same universities.

**Methods:**

Data from 2007–13 from the UK Medical Education Database (UKMED) were available for graduates of gateway and SEM courses at three UK medical schools. Outcome measures were passing an entry exam on the first attempt, Annual Review of Competency Progression (ARCP) outcome and being offered a level one training position from the first application. The univariate analysis compared the two groups. Logistic regressions, predicting outcomes by course type, controlled for attainment on completion of medical school.

**Results:**

Four thousand four hundred forty-five doctors were included in the analysis. There was no difference found in the ARCP outcome between gateway and SEM graduates. Gateway graduates were less likely to pass their first attempt at any membership exam than graduates of SEM courses (39% vs 63%). Gateway graduates were less likely to be offered a level 1 training position on their first application (75% vs 82%). Graduates of gateway courses were more likely to apply to General Practitioner (GP) training programmes than SEM graduates (56% vs 39%).

**Conclusions:**

Gateway courses increase the diversity of backgrounds represented within the profession and importantly the number of applications to GP training. However, differences in cohort performance are shown to continue to exist in the postgraduate arena and further research is required to ascertain the reasons for this.

**Supplementary Information:**

The online version contains supplementary material available at 10.1186/s12909-023-04179-3.

## Background

Worldwide, there is a concern that doctors do not reflect the socio-demographic diversity of the patients they serve[1, 2]. The 2013 *UK State of the Nation* report highlighted the lack of diversity within the medical profession [3]. Subsequently, the Medical Schools Council (MSC) led a major review, S*electing for Excellence*, which confirmed that a ‘key issue for medicine … is the unrepresentative number of students from a lower socioeconomic background currently at medical school’ [4]. This is a well-recognised problem and a UK Government priority [5, 6]. In addition, in the United Kingdom (UK), there is a shortage of General Practitioners (GPs) serving our population, which is expected to increase [7]. Widening Participation (WP) to medicine initiatives, particularly gateway courses, could go some way to address these concerns. These programmes typically consist of an added year of medical education with specialist student support front-loaded onto the existing five-year medical curriculum [8]. Once complete, students typically join pre-existing five-year programmes with continued tailored support [9]. Typically, all additional support ceases on graduation when students gain provisional registration with a licence to practise. They are designed to attract and support students from educationally and socially disadvantaged backgrounds to study medicine [5, 7, 10]. Their overarching purpose is to drive the NHS to be representative and understanding of its current and future patients (healthcare provision) and to improve social mobility [11]. Gateway courses are increasing in number, in 2018 the MSC listed 10 such courses in their medical school entry requirement documentation; in 2023 they listed 19 [5, 12, 13]. This study is the first to compare postgraduate outcomes in doctors trained on a gateway course with those from traditional standard entry to medicine (SEM) courses in the UK, comparing their progression through training and specialty application choices.

Established gateway courses successfully recruit students from underrepresented backgrounds, with the majority of students progressing and graduating as doctors [10, 14]. Curtis and Smith (2020) found that gateway students demonstrate reduced attainment on entry compared to SEM students [10]. Despite the difference reported, gateway courses are shown to support most students in succeeding at medical school. The gap between the attainment of gateway students and that of their standard entry peers reduces throughout their undergraduate journey [10, 14, 15]. However, a gap remains on graduation. It is important to understand that the impact of social and economic disadvantage does not cease simply because students have attained a place at medical school [10]. Curtis and Smith (2020) stressed the importance of contextualising the difference seen in undergraduate outcomes between the gateway and SEM cohorts. The authors suggest the frequently seen additional demands on gateway students’ time and the need for institutional change could be factors that prevent gateway students from reaching their potential at medical school [16]. They postulated that additional support raised awareness and implementation of institutional responsibility could support and optimise their progression [10].

Although the success of the gateway courses can be seen as completing a medical degree, a more pragmatic and long-term view of success may be seen as graduating doctors who successfully navigate through their postgraduate training. There would be little benefit in widening participation to a medical degree if graduates were not able to progress through training, to support the profession being more representative of the population it serves [11]. It is unclear whether the attainment gap on graduation from medical school continues to diminish, remains the same or widens after this stage [10]. In addition to increasing representation, there is also a need to increase the number of doctors in underserved and, often, socially deprived areas of the UK [7, 11, 17]. Students on gateway courses frequently originate from such areas and evidence from the UK and United States (US) supports the notion that a doctor’s childhood background is a strong predictor of the population they will subsequently serve [18].

**Fig. 1:**
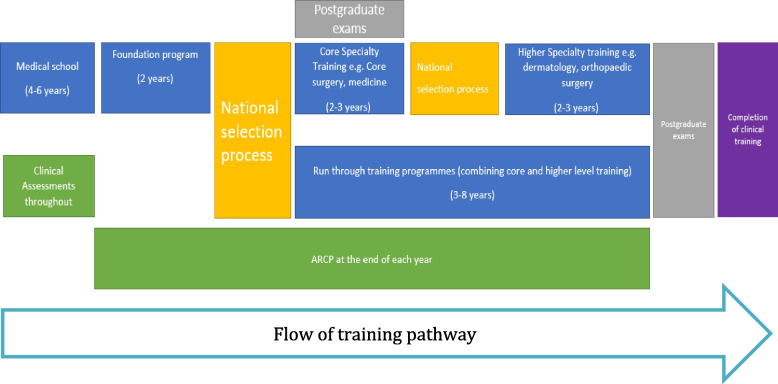
A diagrammatic representation of progression through a clinical career pathway as a doctor in the United Kingdom

Alongside choices doctors make about their career trajectory, there are several milestones that they must reach to continue their progression as outlined in Fig. 1.

The first stage of a doctor’s postgraduate training in the UK is the foundation programme consisting of two years of broad-based training in various specialties. In the foundation programme, each medical trainee’s progress is reviewed at an Annual Review of Competency Progression (ARCP). The rating at ARCP is based on a portfolio of evidence collated in the e-portfolio, including reviews from supervisors. Passing this summative component is a prerequisite to progression to the next stage of training [19, 20]. In their second year of foundation training or post-completion of foundation, training doctors can apply to a national selection process to progress into core/run-through specialty training for which they may or may not be offered a training position in one or more of the specialities they applied to. These specialties will require them to sit membership exams to progress further and will often reward applicants for taking the exams before the interview (although they are not a compulsory component at this stage).

This study is a continuation of the undergraduate outcomes previously reported by Curtis & Smith [10] for these cohorts. The current study aims to compare their postgraduate outcomes using, ARCP outcome, membership exam first attempt pass rate and offer of a level one position in their given speciality choice [10]. These variables are independent of one another although closely related.

## Methods

### Data

Data were collected from doctors who had graduated from a gateway or SEM course at three medical schools (Norwich, King’s College London and Southampton medical schools). These medical schools were chosen as they run the most established gateway courses sharing the same content for years 1–5 and have a similar science/professionalism balance in the first year. As such, sufficient data were available regarding their postgraduate outcomes. Data were collected for the cohorts starting between 2007 and 2013 via the UK Medical Education Database (UKMED) and they were comparable. This timeframe was based on the commencement date contained in The Higher Education Statistics Agency (HESA) student record [26] and sufficient time post-graduation to collect meaningful data on progression. The selection process and relevant exclusion criteria are demonstrated in Fig. 1 below.

**Fig. 2:**
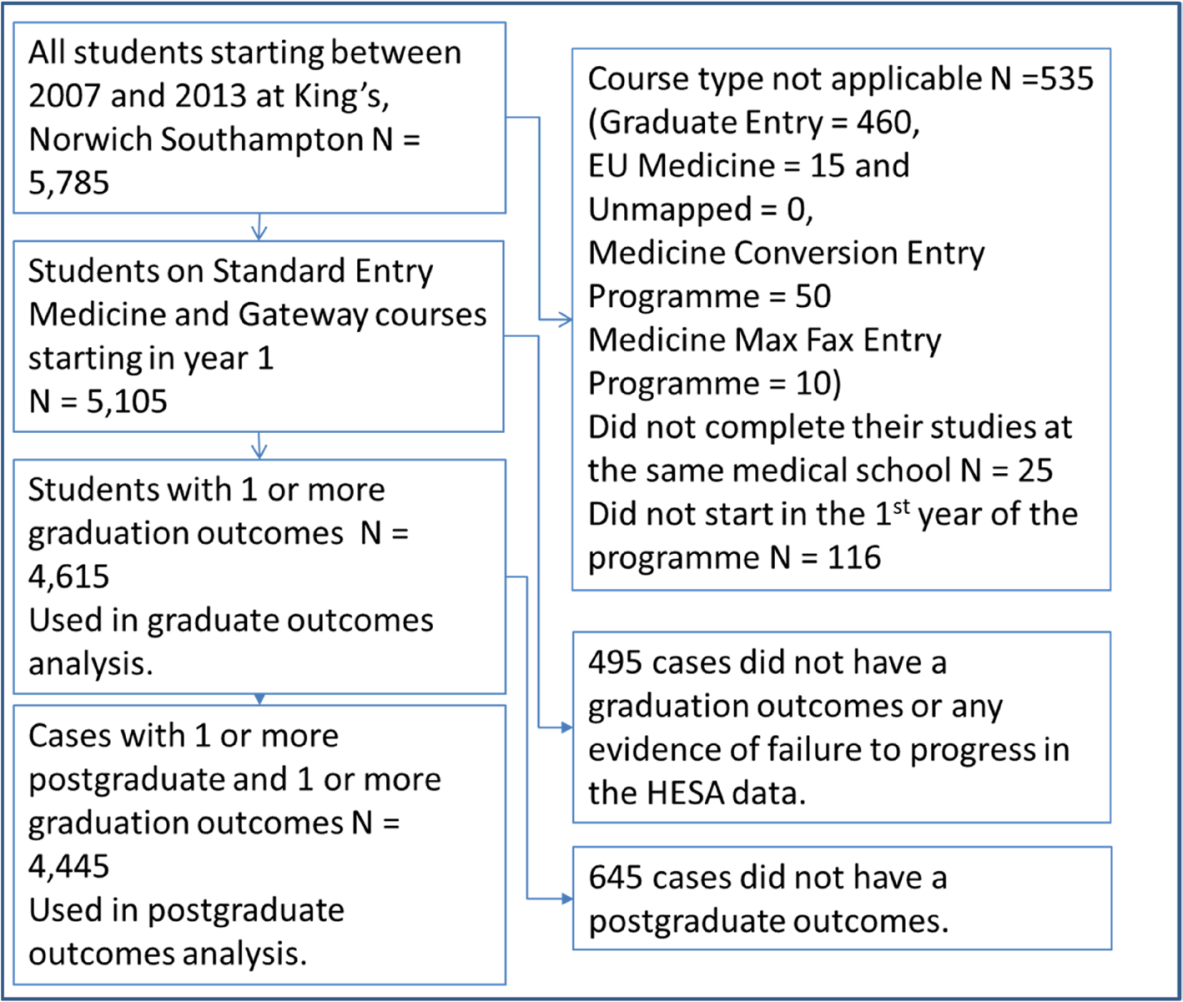
Flow of data through the study with the cases removed by exclusion criterion

Figure 2 shows how the 4445 participants were selected for postgraduate analysis. 20.2% (*N* = 740) of the gateway cases had no graduate outcomes compared to 340 7.9% (*N* = 4,370) of SEM cases (*P* < 0.001). This is due to the higher attrition on the gateway course noted by Curtis and Smith [10]. There was no difference in the availability of outcome measures across the three schools included in the study.

27.6% (*N* = 740) of gateway cases had no postgraduate outcome measures available compared to 10.1% (*N* = 4,370) of SEM cases (*P* < 0.001).

## Measures

### ARCP summary measures

#### ARCP outcome

ARCPs were introduced into foundation training in 2012 [24]. These foundation ARCP outcomes were collected for the training years 2013 through 2020.

The foundation ARCP outcomes have been summarised by calculating if the trainee received one or more of the following outcomes at any point during their foundation training programme:

- a) Foundation programme outcome 3: Has not achieved competencies required to progress, additional training required.
- b) Foundation programme outcome 4: Released from a training programme with or without specified competencies.
- c) Foundation programme outcome 5: Incomplete evidence provided.

The ARCP outcomes awarded during specialty training [21] have also been summarised by calculating if the trainee was awarded one or more of the given outcomes during their time in specialty training. These outcomes were collected for the training years 2013 through 2020. Appendix Table A2 lists the specialties these outcomes referred to.

- a) Specialty programme outcome 2: May progress but requires specific/targeted training to achieve certain competencies
- b) Specialty programme outcome 3: Has not achieved competencies required to progress, additional training required
- c) Specialty programme outcome 4: Released from a training programme with or without specified competencies
- d) Specialty programme outcome 5: Incomplete evidence provided

### Passing the first attempt at any membership exam

No individual Royal College membership exam had sufficient cases to make comparisons across the two course types, therefore, a consolidated variable called “pass on first attempt at any membership exam” was used. Appendix Table A1 lists the exams included in this measure. These exams were sat between 1st August 2013 and 31st July 2020.

**Table 1:** Summary of demographic data (*N* = 4,445)

		**Gateway**	**SEM**	**Test of association**	
**Factor**	**Category**	**N (%)**	**N (%)**	**Χ**^**2**^	**P – Bonferroni correction applied**
Sex	Male	235 (44.4%)	1680 (43.0%)	0.309	1
	Female	295 (55.6%)	2230 (57.0%)		
Ethnicity groups	BME	350 (65.6%)	1560 (39.9%)	138.558	< 0.001
	White	155 (29.5%)	2210 (56.6%)		
	Missing	25 (4.9%)	165 (3.6%)		
Disability last	No known disability recorded	415 (77.8%)	3430 (87.7%)	38.617	< 0.001
	1 or more recorded by HESA	120 (22.2%)	480 (12.3%)		
School Type (HESA)	From state-funded school	510 (96.2%)	2875 (60.4%)	262.841	< 0.001
	Privately funded school	15 (2.6%)	1040 (26.6%)		
	Missing	5 (1%)	505 (13%)		
Parental education (higher education)	No	335 (62.8%)	710 (18.2%)	539.909	< 0.001
	Yes	140 (26.5%)	2800 (71.6%)		
	Unknown	55 (10.7%)	400 (10.3%)		
Participation in local areas (POLAR)	1	45 (8.8%)	140 (3.6%)	289.544	< 0.001
	2	95 (17.7%)	270 (6.9%)		
	3	150 (28.6%)	505 (12.9%)		
	4	125 (23.9%)	885 (22.6%)		
	5	110 (20.9%)	1645 (42.1%)		
	Missing	0 (0.2%)	470 (12%)		
Index of multiple deprivation (IMD)	1—Most deprived	200 (38.0%)	225 (5.7%)	721.006	< 0.001
	2	115 (21.4%)	415 (10.6%)		
	3	100 (19.2%)	665 (17.0%)		
	4	55 (10.2%)	855 (21.9%)		
	5—Least deprived	60 (11.1%)	1285 (32.9%)		
	Missing	0 (0.2%)	465 (11.9%)		
Socioeconomic classification (SEC)	Semi-routine and routine occupations	140 (26.5%)	305 (7.8%)	316.515	< 0.001
	Lower supervisory and technical occupations	20 (3.9%)	70 (1.8%)		
	Small employers and own account workers	45 (8.6%)	205 (5.3%)		
	Intermediate occupations	60 (11.1%)	350 (9.0%)		
	Managerial and professional occupations	205 (38.3%)	2,820 (72.1%)		
	Unknown	60 (11.5%)	160 (4.0%)		
UCAT Bursary	No	360 (67.7%)	3700 (94.6%)	429.497	< 0.001
	Yes	170 (32.3%)	210 (5.4%)		

### Z-score for the first attempt at any membership exam

The score relative to the pass mark was calculated for each exam for which the General Medical Council GMC held an overall score. This was converted to a Z-score by obtaining the mean and standard deviation of all cases in UKMED (not just those in this research extract) for the given exam and date.

### Offered any level 1 position from the first application made

This measure looks across all applications to specialty training programmes made in the first year in which the graduate applied and whether they were offered a place on any training programme. They may have been offered more than one and they may not have accepted the position. These applications were made for programmes starting between 2012 and 2020.

### Recruitment measures

After doctors complete the foundation programme or equivalent training, they are eligible to apply for higher level training referred to as ‘specialty training’. Some of these training programs have further prerequisites such as examinations/other training that must be completed.

Specialty programmes are applied for at a national level and successful applicants are granted a ‘training number’ coinciding with the specialty training programme they have successfully been recruited for.

This measure was divided into:

• Applied to – the specialty applied for on the first specialty application to programmes between 2012 and 2020. Applications to multiple specialties in the same year are possible [22].
• Offered – whether the applicant was offered a place on the programme based on their 1^st^ application. Only considered where N > 50 for gateway applicants to the programme.

## Results

Demographic data (Table 1) supports previous work showing intersectionality between gateway course students and those from BME backgrounds. Gateway graduates were also more likely to have one or more disabilities, have a lower IMD and come from state-funded schools.

### Postgraduate outcomes

Table 2 gives the univariate comparisons between graduates of SEM courses and those from gateway courses for each measure.

**Table 2:** Univariate comparisons of postgraduate outcomes between SEM and gateway graduates

Postgraduate outcome	Gateway				SEM				
	**%**	**N**	**Lower 95% CI**	**Upper 95% CI**	**%**	**N – HESA rounding applied**	**Lower 95% CI**	**Upper 95% CI**	**P—Bonferroni correction applied**
**Summary measures**									
Passed 1st Membership exam sat	39%	195	33%	46%	63%	2,270	61%	65%	< 0.001
Appointable to any level 1 position from 1st application made	87%	335	83%	90%	90%	2,890	89%	91%	1
Offered any level 1 position from 1st application made	75%	345	70%	79%	82%	2,950	81%	84%	0.019
**ARCP summary measures—foundation**									
Foundation programme outcome 5	18%	530	15%	21%	18%	3,895	17%	19%	1
Foundation programme outcome 3	2%	530	1%	3%	2%	3,895	1%	2%	1
Foundation programme outcome 4	0%	530	0%	1%	0%	3,895	0%	0%	1
**ARCP summary measures – specialty**									
Specialty programme outcome 5	21%	195	16%	28%	31%	2,050	29%	33%	0.195
Specialty programme outcome 2	12%	195	8%	17%	10%	2,050	8%	11%	1
Specialty programme outcome 3	7%	195	4%	12%	7%	2,050	6%	8%	1
Specialty programme outcome 4	1%	195	0%	4%	2%	2,050	1%	2%	1
**Recruitment measures—applied to**									
Applied—Acute Care Common Stem—Emergency Medicine	4%	345	2%	6%	6%	2,940	5%	7%	1
Applied—Clinical Radiology	3%	345	2%	6%	5%	2,940	4%	6%	1
Applied—Core Anaesthetics Training	10%	345	8%	14%	14%	2,940	13%	15%	1
Applied—Core Medical Training	11%	345	8%	14%	18%	2,940	17%	20%	0.017
Applied—Core Psychiatry Training	5%	345	3%	8%	6%	2,940	5%	7%	1
Applied—Core Surgical Training	17%	345	13%	21%	15%	2,940	14%	16%	1
Applied—General Practice	56%	345	51%	61%	39%	2,940	38%	41%	< 0.001
Applied—Obstetrics and Gynaecology	6%	345	4%	9%	5%	2,940	5%	6%	1
Applied—Ophthalmology	3%	345	2%	5%	3%	2,940	2%	3%	1
Applied—Paediatrics	6%	2,940	5%	7%	5%	345	3%	8%	1
Applied—Internal Medicine Training	7%	2,940	7%	8%	6%	345	4%	9%	1
Applied—Neurosurgery	2%	345	1%	4%	1%	2,940	1%	2%	1
Applied—Public Health Medicine	1%	345	0%	3%	2%	2,940	1%	2%	1
Offered – Core Surgical Training	45%	60	33%	58%	61%	445	56%	65%	0.685
Offered—General Practice	79%	190	72%	84%	84%	1,160	81%	86%	1

These comparisons did not suggest there were differences in ARCP outcomes during foundation training. The percentage of trainees being awarded outcomes 3, 4 or 5 during foundation training did not differ across the two course types. Similarly, there was no difference in ARCP outcomes 5, 2,3 and 4 during specialty training across the two groups. Four differences were found, and these were further explored using multivariate analysis (table 3 below):

**Table 3:** Logistic regressions predicting post-graduate outcomes by course type controlling for attainment on completion of medical school

	**Variable**	**B**	**SE**	**P**	**Exp(B)**	**95% C.I. for EXP(B)**	
						**Lower**	**Upper**
Model 1—Passed 1st Membership exam sat N = 2,450 Overall Model Χ^2^ = 49.773. *P* < 0.001	Final School			0.014			
	King's	0.213	0.098	0.030	1.238	1.021	1.501
	Norwich	-0.064	0.116	0.579	0.938	0.747	1.177
	Gateway	-0.959	0.153	0.000	0.383	0.284	0.517
	(Constant)	0.452	0.078	0.000	1.572		
Model 2—Passed 1st Membership exam sat *N* = 2,450 Overall Model Χ^2^ = 422.070. *P* < 0.001	Final School			0.014			
	King's	0.136	0.106	0.201	1.146	0.930	1.411
	Norwich	-0.197	0.125	0.115	0.821	0.643	1.049
	Gateway	-0.700	0.165	0.000	0.496	0.359	0.686
	EPM normal deviate	1.110	0.063	0.000	3.033	2.678	3.435
	(Constant)	0.460	0.084	0.000	1.584		
Model 1- Offered on first application round- *N* = 3,280 Overall Model Χ^2^ = 27.441 *P* < 0.001	Final School			0.000			
	King's	0.402	0.104	0.000	1.496	1.219	1.835
	Norwich	0.356	0.125	0.004	1.427	1.117	1.824
	Gateway	-0.461	0.135	0.001	0.631	0.484	0.821
	(Constant)	1.320	0.080	0.000	3.743		
Model 2—Offered on the first application round 3,280 Overall Model Χ^2^ = 48.944 *P* < 0.001	Final School			0.000			
	King's	0.396	0.105	0.000	1.486	1.210	1.824
	Norwich	0.342	0.126	0.006	1.408	1.100	1.800
	Gateway	-0.358	0.137	0.009	0.699	0.535	0.914
	EPM normal deviate	0.265	0.057	0.000	1.303	1.164	1.458
	(Constant)	1.315	0.080	0.000	3.724		
Model 1—Applied to – General practice *N* = 3,270 Overall Model Χ^2^ = 35.658 *P* < 0.001	Final School			0.281			
	King's	-0.016	0.084	0.848	0.984	0.835	1.160
	Norwich	0.123	0.099	0.212	1.131	0.932	1.373
	Gateway	0.662	0.115	0.000	1.938	1.546	2.430
	(Constant)	-0.447	0.067	0.000	0.640		
Model 2—Applied to—General practice *N* = 3,270 Overall Model Χ^2^ = 61.242 *P* < 0.001	Final School			0.241			
	King's	-0.007	0.084	0.938	0.993	0.842	1.172
	Norwich	0.139	0.099	0.163	1.149	0.946	1.395
	Gateway	0.574	0.117	0.000	1.775	1.412	2.232
	EPM normal deviate	-0.224	0.044	0.000	0.799	0.733	0.872
	(Constant)	-0.436	0.068	0.000	0.647		
Model 1—Applied to – Core Medical Training *N* = 3,270 Overall Model Χ^2^ = 18.562 *P* < 0.001	Final School			0.066			
	King's	-0.079	0.110	0.470	0.924	0.745	1.145
	Norwich	0.186	0.125	0.136	1.204	0.943	1.537
	Gateway	-0.615	0.181	0.001	0.541	0.379	0.770
	(Constant)	-1.505	0.086	0.000	0.222		
Model 2—Applied to—Core Medical Training *N* = 3,270 Overall Model Χ^2^ = 27.056 *P* < 0.001	Final School			0.069			
	King's	-0.089	0.110	0.418	0.915	0.738	1.135
	Norwich	0.175	0.125	0.161	1.191	0.933	1.521
	Gateway	-0.548	0.182	0.003	0.578	0.405	0.826
	EPM normal deviate	0.166	0.057	0.004	1.180	1.056	1.320
	(Constant)	-1.520	0.087	0.000	0.219		

Graduates of gateway programmes were:

- Less likely to pass their first attempt at any membership exam than graduates of SEM courses: 39% compared to 63% passing their first exam. Z-scores of the score relative to “pass for the first attempt” were also compared, for gateway graduates the mean was -0.355 *N*= 175, for SEM graduates the mean was 0.225 *N* = 1930. Cohen’s *d* = 0.627,
- Less likely to be offered a level 1 training position on their first application than SEM graduates: 75% of gateway graduates were offered a position versus 82% of SEM graduates (*P* = 0.019).
- More likely to apply to GP training programmes than graduates from SEM programmes: 56% compared to 39% (*P*< 0.001). There was no statistically significant difference in whether the graduates from each programme were offered places on a GP training programme (79% for gateway and 84% for SEM (*P* =1).
- Less likely to apply to core medical training (two-year core training programme between foundation and higher specialty registrar medical training as per figure 1) than graduates from SEM programmes:11% compared to 18% (*P* = 0.017).

Graduates of gateway courses were:

• 0.38 times less likely to pass their first attempt at a membership exam compared to graduates of SEM courses. This difference decreased to 0.50 times less likely when performance at the end of medical school (EPM normal deviate score) is adjusted for in model 2.
• 0.63 times less likely to be offered a place on any level 1 training program in their first year of applying, this decreases to 0.69 when adjustment for performance medical school (EPM normal deviate) is included in the model.
• 1.94 times more likely to apply to GP training (Model 1). Once attainment on exit from medical school is included (Model 2) this difference is similar at 1.76. Graduates with a lower EPM deviate score were less likely to apply to GP training.

Graduates of gateway courses were approximately 0.55 times less likely to apply to Core Medical Training, regardless of whether the model includes the EPM normal deviate score.

## Discussion

### Annual review of competence progression (ARCP)

The ARCP process is a multifaceted review designed to ensure postgraduate trainees’ progression is managed consistently. It is theoretically designed to be an objective marker of readiness to progress [20]. However, concerns regarding its validity have been raised [23]. The results of this study show that there is no significant difference in attainment of ARCP between the gateway and SEM graduates but that there is a difference in terms of first-time exam pass rate. Postgraduate exams are also designed to measure the readiness of a trainee to progress in their chosen specialty [24]. Although they are testing different types of knowledge, it is surprising that trends between the two metrics are not more closely aligned.

### First-time exam pass rates

Graduates from gateway courses in the UK are less likely to pass their first attempt at a professional exam. This difference is reduced but still present after controlling for attainment on entry. Previous work showed differences in attainment between these two groups lessen during their undergraduate journey [3]. The Cohen’s d for the difference in Z-score of score relative to pass of 0.627 is similar to the difference between the two groups on exit from medical school. In previous work, SEM students had higher EPM scores (Cohen’s d = 0.616) and Prescribing Safety Assessment (PSA) scores (Cohen’s d = 0.653) [10].

The demands of life alongside challenging and demanding jobs that leave little time for preparation are likely to contribute to exam failure. Previous work has raised concerns about equity in postgraduate examinations, such as challenges with the recruitment of diverse examiners for practical exams [25]. Research does exist to support the relationship between passing postgraduate exams and improved patient care in other countries. One study from the US has identified an inverse relationship between the postgraduate medical exam “Step 2 CK USMLE” and patient mortality after controlling for other factors [26]. Work by Hutchinson and colleagues highlighted that there is little research on the validity of postgraduate exams in the UK [27]. Examination validity may be of importance when deciding on how best to support trainees from WP backgrounds.

Similar findings occurred in studies investigating the difference in exam pass rates of ethnic minority doctors with a 12% difference between doctors who identify as white and those who identify as ethnic minority doctors in the UK. This gap widened to 30% in doctors who trained overseas [28]. As previously mentioned, intersectionality between WP and ethnic minority groups exists. A meta-analysis by Woolf et al. found that widespread differences exist in academic performance between “white” and “non-white” medical students and doctors. This existed nationally in a range of exams. This study adds to the body of evidence that brings into question whether postgraduate medical exams are inclusive [29].

### Attaining a level 1 training programme in the first year of applying

Graduates of gateway courses are 0.63 less likely to be offered a place on any level 1 training programme in their first year of applying. This is a competitive national process whereby applicants submit portfolios followed by sitting exams for certain specialties. From that point, applicants undergo an interview for most specialties, which is also a competitive process [30]. It is worth noting that the interview/selection process would not directly identify what kind of undergraduate pathway the doctor undertook to assessors.

There are many potential reasons why WP graduates may fare worse in this process. Table one reveals that WP graduates are less likely to have had a private school education. The Sutton Trust and Social Mobility 2019 report published data that showed *“The prospects of those educated at private schools remain significantly brighter than their peers”.* The paper also identified that students who attend such schools are often grouped with those from a similar background and gain skills in creating networks that are supportive in getting ahead later in life [31].

### Choices

This is the first study to provide insight into the progression and choices made by graduates of UK gateway courses as compared to their SEM counterparts. One significant difference was that graduates of a gateway course are 1.76 times more likely to apply for GP training after attainment at medical school is controlled for.

This builds on the previous body of literature which suggests that doctors from a WP background are more likely to apply for GP training, despite similar competition ratios between specialties [32]. For example, in 2013 the competition ratio for entry to GP training was 2.3 whilst the ratio for Core Medical Training (CMT) 2.6. For context the competition ratio for a level one position on radiology pathway was 4.1 and cardiothoracic surgery pathway was 11.4 [33]. Many potential explanations for this exist, including that these doctors want to serve their community directly, high costs associated with other training programmes, and the perceived benefits of GP lifestyle regarding family life [34].

Despite the Government’s position in 2015 that 5,000 more GPs were needed in the United Kingdom by 2020, further reductions seem to have occurred [18]. The results from this study support the suggestion from previous literature that widening participation to medicine may be one method of tackling GP shortages in the UK [18, 35] highlighting the additional importance of gateway courses in NHS workforce planning.

Although doctors from gateway courses preferentially choose GP training, a similar proportion are applying to most other specialties as their SEM counterparts and are being offered posts. The exception to this is CMT (now called internal medicine training – IMT). Graduates of gateway courses were approximately 0.55 times less likely to apply to CMT, regardless of whether the model controlled for attainment. The exception of CMT may be due to a multitude of reasons. A 2013 survey of CMT trainees in the UK identified several areas of concern including three areas deemed “urgently requiring attention” [36].

## Limitations

This study has identified similarities and differences in the choices and outcomes of gateway and SEM postgraduates. However, it has not delineated the reasons for these trends. To understand this better, further work is needed to discover what motivates these doctors to make the choices they do. Qualitative data exploring the reasons behind individual choices would clarify the reasons for the trends identified here.

Furthermore, the number of graduates from gateway courses remains small and, although well established, relatively new in comparison to the pre-existing standard entry courses, which may affect the student experience. With more feedback and research these courses are likely to improve and become more in tune with student needs. As such their outcomes may change to reflect this and the difference seen in outcomes may reduce as the courses embed.

Acknowledgements of the need for postgraduate support for WP graduates has been highlighted with the new pre-allocation process for foundation training. However, little evidence exists to suggest what support is needed to mitigate the difference in pass rate further along in the training programme and is an area much in need of further research. As a relatively new cohort, many gateway graduates have yet to complete exams and specialty training. A further study of this cohort in several years will reveal more about the long-term progression and career trajectories of these cohorts. Those with an interest in monitoring the postgraduate outcomes of graduates from different courses can do so using the GMC’s progression reports which are published annually [37].

Finally, it is important to highlight that the first-time exam pass rates and first-time offers are not reflective of the entirety of a doctor’s career and that these are short-term outcomes. Exam failure is common in postgraduate exams and there are opportunities to retake them before they become a limiting factor in progression, for example re-sitting the exam up to the maximum number of times permitted. There is not yet enough data available for this cohort to know if this will occur. Once this study’s cohort has reached the end of their postgraduate training, it will be possible to ascertain whether gateway graduates are as successful as SEM graduates in joining the specialist register – obtaining their certificate of completion of training (CCT) [30]. Further research examining differences throughout the doctors’ careers are needed to draw more detailed conclusions with CCT being the ultimate outcome.

## Application to policy and practice

Concern regarding the number of doctors in general practice is rising. Alongside this is a concurrent acceptance that the variation in demographics of doctors should reflect that of the general population [4, 6, 18]. This study has supported previous literature in showing that doctors from gateway courses have a preference for general practice [32, 38, 39]. It has previously been suggested that increasing the number of gateway places at medical schools is a reasonable option to combat the lack of GPs in the face of growing need and this study’s findings support that notion [40].

## Conclusions

The results of this study provide important initial insights into the outcomes for graduates of gateway courses. These findings highlight some of the national benefits such as increasing representativeness within the profession and increased applications to GP training. They also raise more questions that need addressing such as identifying appropriate support provisions and the need to explore potential systemic inequalities in the training programmes and postgraduate exams which are necessary milestones to progression. Furthermore, the results provide some insight into WP graduate choices in comparison to their SEM counterparts. Following this cohort further will likely allow more conclusions to be drawn about progression and choices in the future. Closer scrutiny of postgraduate exam outcomes are needed in order to identify whether differences between these groups are rooted in disadvantages experienced by gateway postgraduate doctors.

## Supplementary Information

**Additional file 1.**

## Data Availability

The source of data was the UK Medical Education Database (“UKMED”) UKMEDP38 extract generated on 13^th^ October 2021 and Approved for publication on 20th December 2018. The final extract was produced after the report had been approved as we re-ran the analyses with an additional year’s worth of data. We are grateful to UKMED for the use of these data. However, UKMED bears no responsibility for its analysis or interpretation. The data includes information derived from that collected by the Higher Education Statistics Agency Limited (“HESA”) and provided to the GMC (“HESA Data”). Source: HESA Student Record [2002/2003 and 2019/2020] Copyright Higher Education Statistics Agency Limited. The Higher Education Statistics Agency Limited makes no warranty as to the accuracy of the HESA Data, and cannot accept responsibility for any inferences or conclusions derived by third parties from data or other information supplied by it. Applications to view the data can be made to UKMED. The data in UKMED are anonymised before use.( process available here: UKMED Research Process) The datasets used and/or analyzed during the current study available from the corresponding author on reasonable request.

## Abbreviations

BMA: British medical association
BME: Black and Minority Ethnic
CMT: Core Medical Training
IMT: Internal Medicine Training
EPM: Educational performance measure
GMC: The general medical council
HE: Higher education
HESA: Higher education statistics agency
IMD: Index of multiple deprivation
MMIs: Multiple-mini interviews
MRCPUK: Membership of the royal colleges of physicians of the United Kingdom
MSC: Medical schools council
NHS: National health service
PSA: Prescribing safety assessment
SEM: Standard entry medicine
SES: Socioeconomic status
SJT: Situational judgement test
UK: United Kingdom
UKMED: UK medical education database
WP: Widening participation
