# Supplementary material for: How do the post-graduation outcomes of students from gateway courses compare to those from standard entry medicine courses at the same medical schools?
Source: BMC Med Educ. 2023 May 2;23:298. doi: 10.1186/s12909-023-04179-3 (PMC10152708; doi:10.1186/s12909-023-04179-3)
Supplement: Supplementary file 1 — Additional file 1. [file 12909_2023_4179_MOESM1_ESM.docx]

### Supplementary Material

### Appendix A1 Exams included when calculating passed first college exam sat

| **Exam** | **N SEM – HESA rounding** | **N Gateway – HESA rounding** |
| --- | --- | --- |
| First FRCR Clinical Radiology: Anatomy_First FRCR Clinical Radiology: Physics | 45 | 5 |
| FPH FPH Part A | 5 | 0 |
| FSRH MFSRH Part 1 | 5 | 0 |
| ICBSE DOHNS MCQ | 0 | 0 |
| ICBSE MRCS - Part A - MCQ | 430 | 40 |
| ICBSE MRCS - Part B - OSCE | 5 | 0 |
| MRCP MRCP Part 1 | 880 | 60 |
| MRCP Paces_MRCP Part 2 | 0 | 0 |
| MRCP Part 1_MRCP Part 2 | 5 | 0 |
| MRCP SCE Geriatric Medicine | 0 | 0 |
| MRCPCH Foundation of Practice (FOP)_MRCPCH Theory and Science (TAS) | 85 | 5 |
| RCGP MRCGP AKT | 355 | 45 |
| RCGP MRCGP CSA | 0 | 0 |
| RCoA FRCA Primary MCQ | 85 | 5 |
| RCOEM FRCEM Primary | 65 | 5 |
| RCOEM MRCEM Part A | 30 | 0 |
| RCOG MRCOG Part 1 | 95 | 15 |
| RCOG MRCOG Part 2 | 5 | 0 |
| RCOphth FRCOphth Part 1 | 30 | 5 |
| RCPath FRCPath Part 1 Histopathology | 5 | 0 |
| RCPATH FRCPath Part 1 Histopathology | 0 | 0 |
| RCPCH MRCPCH Applied Knowledge in Practice (AKP) | 5 | 0 |
| RCPCH MRCPCH Foundation of Practice (FOP) | 60 | 5 |
| RCPCH MRCPCH Theory and Science (TAS) | 10 | 0 |
| RCPsych RCPsych Paper 1 | 0 | 0 |
| RCPsych RCPsych Paper A | 70 | 5 |
| RCPsych RCPsych Paper B | 5 | 0 |
| RCR First FRCR Clinical Radiology: Anatomy | 0 | 0 |

### Appendix A2 Specialties with ARCP outcomes

| **Specialty group** | **N SEM – HESA rounding** | **N Gateway– HESA rounding** |
| --- | --- | --- |
| ACCS | 130 | 15 |
| ACCS_Core Anaesthetics Training | 10 | 0 |
| ACCS_Core Surgical Training | 0 | 0 |
| ACCS_Emergency Medicine | 25 | 0 |
| ACCS_General Practice | 5 | 0 |
| ACCS_Paediatrics and Child Health | 0 | 0 |
| Broad Based Training_General Practice | 0 | 0 |
| Broad Based Training_Paediatrics and Child Health | 0 | 0 |
| Core Anaesthetics Training | 130 | 10 |
| Core Anaesthetics Training_Core Medical Training | 0 | 0 |
| Core Anaesthetics Training_Core Surgical Training | 0 | 0 |
| Core Anaesthetics Training_Emergency Medicine | 0 | 0 |
| Core Anaesthetics Training_General Practice | 0 | 0 |
| Core Medical Training | 315 | 15 |
| Core Medical Training_Core Psychiatry Training | 0 | 0 |
| Core Medical Training_Core Surgical Training | 0 | 0 |
| Core Medical Training_General Practice | 20 | 0 |
| Core Medical Training_Obstetrics and Gynaecology | 0 | 0 |
| Core Medical Training_Pathology | 5 | 0 |
| Core Medical Training_Radiology | 10 | 0 |
| Core Psychiatry Training | 95 | 10 |
| Core Psychiatry Training_General Practice | 0 | 0 |
| Core Surgical Training | 220 | 15 |
| Core Surgical Training_General Practice | 5 | 0 |
| Core Surgical Training_Paediatrics and Child Health | 0 | 0 |
| Core Surgical Training_Pathology | 0 | 0 |
| Emergency Medicine | 20 | 0 |
| Emergency Medicine_General Practice | 0 | 0 |
| Emergency Medicine_Paediatrics and Child Health | 0 | 0 |
| General Practice | 720 | 95 |
| General Practice_Obstetrics and Gynaecology | 0 | 0 |
| General Practice_Paediatrics and Child Health | 5 | 0 |
| General Practice_Public Health | 0 | 0 |
| General Practice_Radiology | 0 | 0 |
| Obstetrics and Gynaecology | 100 | 10 |
| Ophthalmology | 25 | 0 |
| Paediatrics and Child Health | 120 | 10 |
| Paediatrics and Child Health_Radiology | 0 | 0 |
| Pathology | 15 | 0 |
| Public Health | 10 | 0 |
| Radiology | 80 | 5 |

1. Some trainees have moved specialty and their outcomes are reported against their entire specialty training, the specialties are listed together where this has occurred.
